# MoO_3_ Nanoparticle Coatings on High-Voltage 5 V LiNi_0.5_Mn_1.5_O_4_ Cathode Materials for Improving Lithium-Ion Battery Performance

**DOI:** 10.3390/nano12030409

**Published:** 2022-01-26

**Authors:** Zong-Han Wu, Jeng-Ywan Shih, Ying-Jeng James Li, Yi-De Tsai, Tai-Feng Hung, Chelladurai Karuppiah, Rajan Jose, Chun-Chen Yang

**Affiliations:** 1 Battery Research Center of Green Energy, Ming Chi University of Technology, New Taipei City 24301, Taiwan; smartindigoboy@gmail.com (Z.-H.W.); yjli@mail.mcut.edu.tw (Y.-J.J.L.); d1161p002@mail.mcut.edu.tw (Y.-D.T.); taifeng@mail.mcut.edu.tw (T.-F.H.); 2 Department of Chemical Engineering, Ming Chi University of Technology, New Taipei City 24301, Taiwan; drexel@mail.mcut.edu.tw; 3 Nanostructured Renewable Energy Materials Laboratory, Faculty of Industrial Sciences and Technology, University Malaysia Pahang, Kuantan 26300, Malaysia; rjose@ump.edu.my; 4 Department of Chemical and Materials Engineering and Green Technology Research Center, Chang Gung University, Taoyuan City 333, Taiwan

**Keywords:** spinel-type LiNi_0.5_Mn_1.5_O_4_, 5 V cathode materials, MoO_3_-coated, high-rate performance, high-energy ball-mill method

## Abstract

To reduce surface contamination and increase battery life, MoO_3_ nanoparticles were coated with a high-voltage (5 V) LiNi_0.5_Mn_1.5_O_4_ cathode material by in-situ method during the high-temperature annealing process. To avoid charging by more than 5 V, we also developed a system based on anode-limited full-cell with a negative/positive electrode (N/P) ratio of 0.9. The pristine LiNi_0.5_Mn_1.5_O_4_ was initially prepared by high-energy ball-mill with a solid-state reaction, followed by a precipitation reaction with a molybdenum precursor for the MoO_3_ coating. The typical structural and electrochemical behaviors of the materials were clearly investigated and reported. The results revealed that a sample of 2 wt.% MoO_3_-coated LiNi_0.5_Mn_1.5_O_4_ electrode exhibited an optimal electrochemical activity, indicating that the MoO_3_ nanoparticle coating layers considerably enhanced the high-rate charge–discharge profiles and cycle life performance of LiNi_0.5_Mn_1.5_O_4_ with a negligible capacity decay. The 2 wt.% MoO_3_-coated LiNi_0.5_Mn_1.5_O_4_ electrode could achieve high specific discharge capacities of 131 and 124 mAh g^−1^ at the rates of 1 and 10 C, respectively. In particular, the 2 wt.% MoO_3_-coated LiNi_0.5_Mn_1.5_O_4_ electrode retained its specific capacity (87 mAh g^−1^) of 80.1% after 500 cycles at a rate of 10 C. The Li_4_Ti_5_O_12_/LiNi_0.5_Mn_1.5_O_4_ full cell based on the electrochemical-cell (EL-cell) configuration was successfully assembled and tested, exhibiting excellent cycling retention of 93.4% at a 1 C rate for 100 cycles. The results suggest that the MoO_3_ nano-coating layer could effectively reduce side reactions at the interface of the LiNi_0.5_Mn_1.5_O_4_ cathode and the electrolyte, thus improving the electrochemical performance of the battery system.

## 1. Introduction

Secondary electrochemical cells, such as lithium-ion batteries (LIBs), are an emerging energy storage technology, widely used in portable electronic devices, battery-powered all-electric vehicles (plug-in electric vehicles or hybrid electric vehicles), and other storage grids [1,2,3,4]. However, the development of high-performance cathode materials is the main bottleneck in the commercial application of LIBs. Conventional cathode materials, such as LiCoO_2_, LiFePO_4_, and LiMn_2_O_4,_ exhibit lower energy densities because of their lower working voltages and discharge capacities [5,6]. Recently, LiNi_0.5_Mn_1.5_O_4_ (LNMO) has gained considerable attention as the next-generation cathode material in LIBs, because of its high operating voltage (Ni^2+^/Ni^4+^ ~4.7 V vs. Li/Li^+^,) [7,8,9], which results in a high theoretical energy density of approximately 650 Wh kg^−1^ [9]. However, this high voltage results in several undesired reactions, such as the decomposition of organic electrolytes and local redox reactions predominantly occurring at surface facets [10]. In particular, when LMNO is charged, Ni^4+^ exhibits extremely high chemical reactivity because of the transfer of electrons between the Ni^4+^ cation in LUMO and the CO_3_^2−^ anion in the solvent [11]. In addition, the other transition metal, Mn^2+^, is directly related to the capacity loss, and its formation is predominantly influenced by surface reactions at the interface of the electrode and electrolyte, instead of disproportionation reactions (2Mn^3+^ → Mn^2+^ + Mn^4+^) [12,13]. Moreover, the cathode–electrolyte interphase (CEI) layer, which is formed because of the surface reactions, allows the passage of Li ions during cycling. Although the CEI layer is beneficial for preventing additional degradation, it may not be preferred for CEI layers that are extremely thick [14,15]. An additional factor that leads to capacity loss is the corrosive dissolution of the cathode when attacked by hydrofluoric (HF) acid. In all LiPF_6_ electrolytes, some degree of salt decomposition occurs, forming LiF and PF_5_ [16]. The traces of H_2_O present in the electrolyte react spontaneously with PF_5_, a strong Lewis acid, resulting in HF formation. The reaction process occurs as described in Equations (1) and (2).

LiPF_6_ ⇄ LiF + PF_5_ (1)

PF_5_ + H_2_O → 2HF + PF_3_O (2)

In attempts to remove the detrimental HF molecules produced in the electrolyte and prevent direct contact of the electrolyte with the active material surface, researchers have coated the cathode surface with oxide materials that are chemically inert in the electrolyte. Numerous oxide-based coating materials have been investigated for LNMO surface modification, including Al_2_O_3_ [17], SiO_2_ [18], V_2_O_5_ [19], CuO [20], ZnO [21], ZrO_2_ [22], and RuO_2_ [23] etc. However, the majority of these materials are electrochemically inactive, i.e., some capacity of the cell is sacrificed when LNMO is coated with the aforementioned oxide materials. Molybdenum trioxide (MoO_3_) is a favorable oxide coating material as compared with these inert metal oxides, as well as fluorides and phosphides, because of its excellent Li-diffusion property, high electrochemical reactivity, and low cost. In recent years, MoO_3_ has been investigated as a new coating material to modify cathode-material surfaces, because MoO_3_ considerably enhances Li ion insertion and extraction properties. It also exhibits a high Li-ion storing capability, because van der Waals forces of attraction can exist in a MoO_3_-layered structure, which results in improved discharge capacity. The MoO_3_-layered structure comprises two MoO_6_ octahedron lattices that share O−O edges. Mo is at the top of the cubic cell, and its unit cell edges are occupied by O atoms. Thus, the MoO_3_-layered structure exhibits a large coordination number of 12 because of the two octahedral lattices, which causes the open structure to enhance Li^+^ ion insertion and extraction properties. In this regard, MoO_3_ structures have a higher Li^+^ ionic diffusivity than other coating materials [24]. For example, Wu et al. [24] proposed a high-energy ball-milling method for incorporating MoO_3_ into Li_1.2_Mn_0.54_Ni_0.13_Co_0.13_O_2_ cathode materials. The results revealed that the initial irreversible capacity can significantly reduce from 81.8 to 1.2 mAh g^−1^, while increasing the MoO_3_ concentration from 0 to 20 wt.%. Because MoO_3_ is an acidic oxide, it can function as an HF scavenger, thereby preventing cathode-material–electrolyte reactions at the cathode surface during high-voltage operations. This characteristic of MoO_3_ improves the electrochemical performance of the material. In another study, the wet-chemical method was used to modify the LiNi_0.5_Co_0.2_Mn_0.3_O_2_ cathode materials with MoO_3_ [25]; the properties of the interface between the cathode materials and electrolytes were improved by the MoO_3_ coating layer, which considerably reduced the side reactions at the interface. The high-rate capability and cycling performance of the LiNi_0.5_Co_0.2_Mn_0.3_O_2_ cathode were significantly improved even for 3 wt.% MoO_3_-coated material. However, no reports have been made regarding surface modification through the coating of 5 V spinel cathode materials with MoO_3_ nanoparticles, and the influence of MoO_3_ layer on the electrochemical performance of 5 V spinel cathode materials remains unclear.

In this study, we investigated the surface modification of 5 V LNMO cathode materials using MoO_3_ nanoparticles, to ascertain its effect on electrochemical performance. As expected, the MoO_3_-coated LNMO cathodes exhibited favorable high-rate performance and long-term cycle life. Furthermore, a three-electrode system comprising a MoO_3_-coated LNMO working electrode, Li_4_Ti_5_O_12_ (LTO) counter electrode, and Li reference electrode was fabricated based on EL-cell configuration to identify the possible capacity loss mechanisms of LTO/LNMO full cells during the cycling process. Surprisingly, LTO/LNMO full cell can achieve a stable long-term cyclability up to 100 cycles at a 1 C rate with an excellent coulombic efficiency of 99.3%.

## 2. Experimental Method

### 2.1. Synthesis of a Pristine LNMO Material

A pristine LNMO electrode material was prepared using a solid-state high-energy ball-milling method. In the first step, the stoichiometric ratios of Li_2_CO_3_ (99.0%, Alfa Aesar, Ward Hill, MA, USA), MnO_2_ (99.0%, Alfa Aesar, Ward Hill, MA, USA), and Ni(OH)_2_ (99.0%, Alfa Aesar, Ward Hill, MA, USA) precursors were separately dispersed in deionized water, and then the sample was homogeneously mixed using a high-energy ball miller (JBM-C020, JUSTNANO, Tainan City, Taiwan) at 3000 rpm for 2 h. The milled suspension was dried at 120 °C using a spray dryer (EYELA SD-1000, Tokyo, Japan). The resultant dry powders were sintered at 900 °C for 10 h and then annealed at 700 °C for 10 h in air. Finally, pristine LiNi_0.5_Mn_1.5_O_4_ (LNMO) powder was obtained.

### 2.2. Preparation of MoO_3_-Caoted LNMO Materials

Different amounts of MoO_3_-coated LNMO (LNMO-MoO_3_-x) materials were prepared using the precipitation method. Initially, a certain amount of (NH_4_)_6_Mo_7_O_24_ 4H_2_O (99.0%, Alfa Aesar, Ward Hill, MA, USA) was dissolved in deionized water and constantly stirred for 3 h; subsequently, pristine LNMO powders were added. The suspension was dried at 80 °C overnight and sintered in air at 500 °C for 5 h in air to obtain LNMO-MoO_3_-x samples. The coating content on LNMO samples was optimized using 1, 2, and 3 wt.% of MoO_3_ layers and named LNMO-MoO_3_-1, LNMO-MoO_3_-2, and LNMO-MoO_3_-3, respectively.

### 2.3. Materials Characterization

The crystallinity properties of the LNMO materials were analyzed using an X-ray diffraction (XRD) spectrometer (BRUKER D2 PHASER, Karlsruhe, Germany) with a Cu Kα radiation source. Micro-Raman spectra were analyzed using a confocal micro-Renishaw spectroscopy system (In Via confocal micro Renishaw, Gloucestershire, UK) with 632-nm He–Ne laser excitation. The morphology of the LNMO samples was observed through field emission scanning electron microscopy (FE-SEM; JSM-7610F Plus, JEOL Ltd., Tokyo, Japan) with electron dispersive spectroscopy (EDS; Oxford X-MaxN, High Wycombe, UK) to determine the elemental compositions; transmission electron microscopy (TEM; JEM-2100, JEOL Ltd., Tokyo, Japan) was also employed, and X-ray photoelectron spectroscopy (XPS; VG Scientific ESCALAB 250, Thermo Fisher, Waltham, MA, USA) was used to survey the valence of elements using XPSPEAK software. The dissolution of transition metal ions was analyzed using an X-ray fluorescent analyzer (BRUKER S2 PICOFOX, Berlin, Germany).

### 2.4. Electrode Preparation and Measurements

The electrochemical measurements of the as-prepared LNMO cathode materials were examined using a two-electrode system (BioLogic BCS-805, Seyssinet, France) by assembling Li/LNMO half cells (CR2032 coin-cell) in an Ar-filled glove box (MBraun LABstar, Garching, Germany) environment. The working electrodes were fabricated using pristine and MoO_3_-coated LNMO materials, Super P, and polyvinylidene difluoride binders with a mass ratio of 8:1:1 in N-methyl pyrrolidinone to form a homogeneous slurry, which was then spread onto aluminum foil and dried in an oven at 120 °C for 12 h. Li sheets (Aldrich, St. Louis, MO, USA) and polyethylene porous membranes were used as the counter electrode and separator, respectively. The carbonate-based organic electrolyte was used by mixing 1 M LiPF_6_ salt in a fluoroethylene carbonate and diethyl carbonate (1:4 *v*/*v*, Merck) solvent. The cycling voltage of the cells varied in the range of 3.5–5.0 V vs. Li/Li^+^ and the current rates were maintained in the range of 0.1–10 C (1 C = 147 mA g^−1^), respectively. In addition, an Autolab PGSTAT302N (Metrohm Autolab B.V., Houten, The Netherlands) potentiostat was used to obtain the electrochemical impedance spectra of these coin cells. To further investigate the full cell, we used a three-electrode battery system (Bio Logic BCS-805, France) comprising pristine LNMO as the working electrode and LTO (Battery Research Center of Green Energy, New Taipei City, Taiwan) as the counter electrode. The anode slurry was prepared by following the same steps as those used for preparing the cathode. Here, LTO was used as the active material. Subsequently, the slurry was coated onto copper foil and dried in an oven at 80 °C for 12 h. A glass fiber membrane (Aldrich) was used as the separator, and the same organic electrolyte was used for this system. For precise alignment of the LNMO/LTO full cell, the diameters of the cathode and anode were fixed as 13 and 15 mm, respectively. The LTO anode was used in the full cell as the limited electrode to calculate the specific capacity at a constant current density and rate of 0.1 C (1 C = 175 mA g^−1^). The voltage range for charge–discharge measurements was fixed at 2.0–3.5 V.

## 3. Results and Discussion

### 3.1. Structural and Morphological Characterization

**Figure 1:**
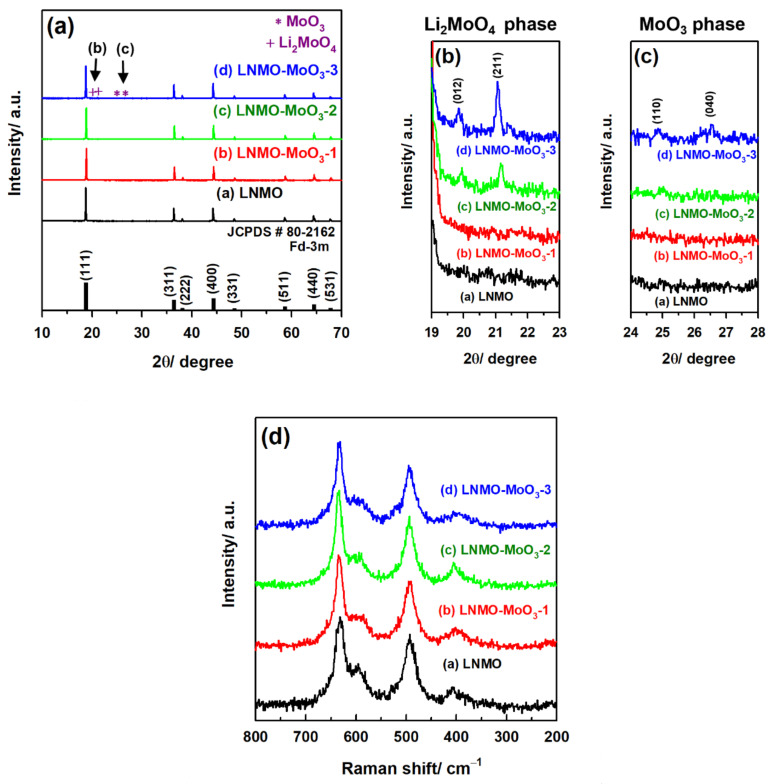
(**a**–**c**) XRD pattern and (**d**) Raman spectra of the LNMO and LNMO-MoO_3_-x samples.

Figure 1a–c depicts the XRD patterns of the LNMO and LNMO-MoO_3_-x samples. All typical peaks clearly matched with those of the LiNi_0.5_Mn_1.5_O_4_ (JCPDS #80-2162) pure crystalline spinel structure, which exhibited the Fd-3m space group with the absence of additional impurity diffraction peaks, such as Li_x_Ni_1-x_O. Because of the high-temperature calcination process, the formation of oxygen vacancy could be compensated for by conducting annealing at 700 °C [19]. The original crystal structure of LNMO was not affected by the MoO_3_ coating element. As indicated by curve a of the XRD results in Figure 1a, a well-crystallized spinel phase (pristine LNMO) could not be observed from the peaks of MoO_3_, whereas two minor peaks were observed in the ranges of 2θ = 19–23° and 2θ = 24–28°, as indicated by curves c and d. This can also be clearly identified by enlarging Figure 1b and c, corresponding to the existence of the Li_2_MoO_4_ and MoO_3_ phases on the LNMO surface, respectively. The peaks at 19.8° and 21° are related to the (012) and (211) planes of the Li_2_MoO_4_ phase (JCPDS #12-0763), and the peaks at 24.8° and 26.6° are related to the (110) and (040) planes of the orthorhombic α-MoO_3_ phase (JCPDS #05-0508). By contrast, the corresponding MoO_3_ peaks were not observed in the XRD pattern of the LNMO-MoO_3_-1 (Figure 1a,c), curve b sample because of the limited coating material.

The diffraction peak intensity of MoO_3_ increased with the increase in concentration of the coating material (2 wt.% and 3 wt.%) [24]. A diffraction peak of Li_2_MoO_4_ was observed. A small amount of Li_2_CO_3_/LiOH impurity formed on the surface of the pristine LNMO materials upon exposure to air (coexistence of CO_2_ and H_2_O). After the sample was annealed at a high temperature, (NH_4_)_6_Mo_7_O_24_ could be decomposed into the MoO_3_ phase, thereby increasing the contact of MoO_3_ with the LNMO surface. This increased contact resulted in an increased reaction with the Li_2_O/LiOH impurity, thus generating Li_2_MoO_4_, which was highly reactive during the Li ion insertion and extraction process [25]. Therefore, the results of XRD analysis indicated the successful coating of MoO_3_ on the LNMO surface.

Furthermore, the spinel LNMO materials exhibited Fd-3m and P4_3_32 space groups for disordered and ordered Ni/Mn metal ion distributions, respectively [26]. However, it was difficult to identify space group details from XRD analysis because the scattering range of Ni and Mn elements is the same [27]. Therefore, micro-Raman spectroscopy was applied. Micro-Raman spectroscopy is an effective analysis technique for the identification of cation ordering because of its sensitivity to crystal symmetry [28]. Figure 1d displays the micro-Raman spectra of LNMO (curve a) and LNMO-MoO_3_-x samples (curves b–d). As displayed by curves a–d in Figure 1d, the peaks observed at 629 cm^−1^ were attributed to the Mn-O stretching vibration of MnO_6_ octahedron, and the peaks observed at 486 and 397 cm^−1^ corresponded to Ni-O stretching vibration in the crystal structure [29]. The peaks observed at 580–600 cm^−1^ were mostly related to the ordered crystal structure with the P4_3_32 space group [30]. The LNMO spectra did not exhibit any clear peak splitting in the range of 580–600 cm^−1^, suggesting that LNMO had a disordered spinel structure. However, in the LNMO-MoO_3_-x samples, these peaks were weaker, indicating that LNMO-MoO_3_-x samples exhibited a highly disordered crystal geometry with the Fd-3m space group. Therefore, the coating process favors the enhancement of the degree of cation disorder.

**Figure 2:**
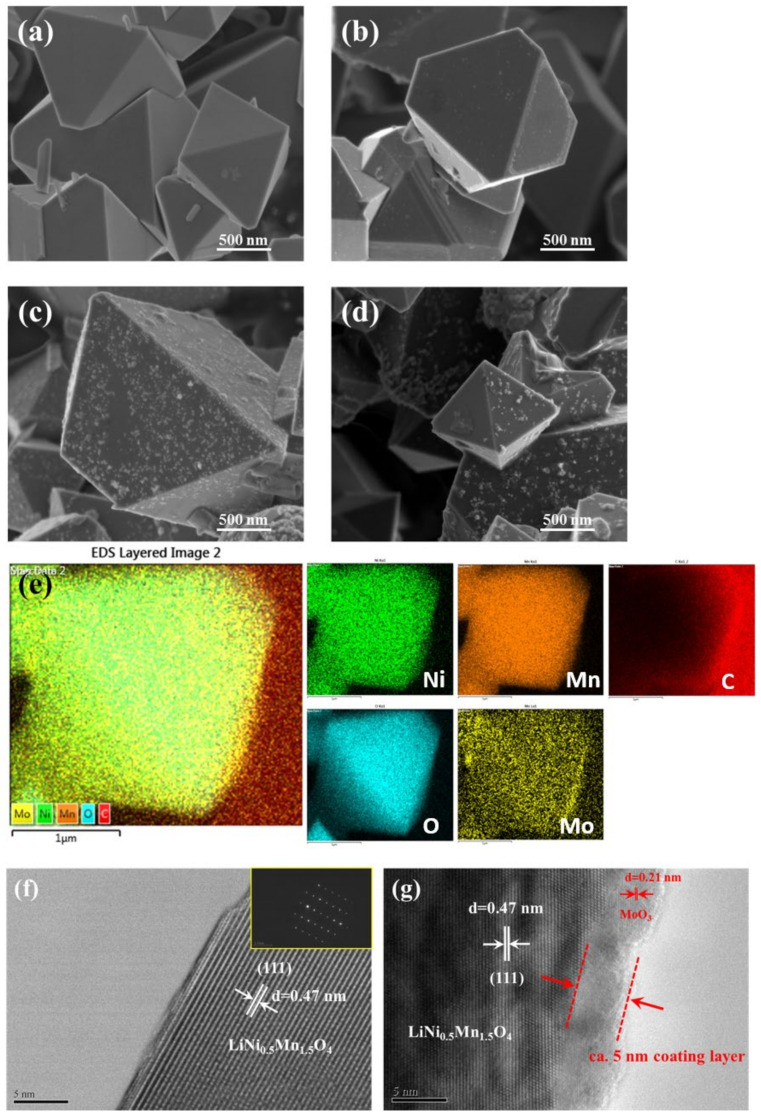
SEM images of pristine LNMO (**a**), LNMO-MoO_3_-1 (**b**) LNMO-MoO_3_-2 (**c**) and LNMO-MoO_3_-3 (**d**); EDS mapping results of LNMO-MoO_3_-2 (**e**); HRTEM images of the pristine LNMO (**f**) and LNMO-MoO_3_-2 (**g**).

Figure 2a–d depict the SEM images for the LNMO and LNMO-MoO_3_-x samples. An octahedral-shaped morphology was observed for all samples, which indicates that the prepared samples exhibited excellent crystallinity. Figure 2a clearly reveals a flat, clean particle surface with a spinel structure for the LNMO sample; by contrast, Figure 2b depicts an irregular sample surface after it was coated with 1 wt.% MoO_3_. A thin layer was clearly deposited on the LNMO surface when the MoO_3_ amount was gradually increased to 2 wt.% (Figure 2c). Further increase in the coating amount caused the LNMO-MoO_3_-3 sample surface to become slightly rougher, indicating that the MoO_3_ layers were uniformly coating the surface of LNMO. Figure 2e illustrates the EDS mapping of the LNMO-MoO_3_-2 sample, which confirms the presence of Ni, Mn, Mo, and O elements in the material. EDS mapping for Mo element indicated that its distribution exhibited the same homogeneity as that observed in the bulk structure, suggesting that the LNMO surface was completely covered by a thin layer of MoO_3_ nanoparticles.

High-resolution TEM (HRTEM) images of LNMO and LNMO-MoO_3_-2 were captured to examine the microstructures of LNMO before and after MoO_3_ coating. The HRTEM (Figure 2f) image of LNMO reveals a flat, clean material surface. A well-crystallized material with high-contrast lattice fringes was observed. The selected area diffraction pattern in the (100) zone revealed a typical Fd-3m space group of the spinel structure. The lattice fringes exhibited an interplanar distance of 0.47 nm, which is closely related to the d-spacing of the (111) planes of LNMO. After coating with MoO_3_, the surface of the LNMO material became rough (Figure 2g). The thickness of the MoO_3_ coating layer on the LNMO particle was approximately 5 nm, and the lattice fringes exhibited an interplanar distance of 0.21 nm, which corresponded to the (141) planes of MoO_3_. However, the parent particle appeared to remain in the spinel LNMO phase even after MoO_3_ coating.

**Figure 3:**
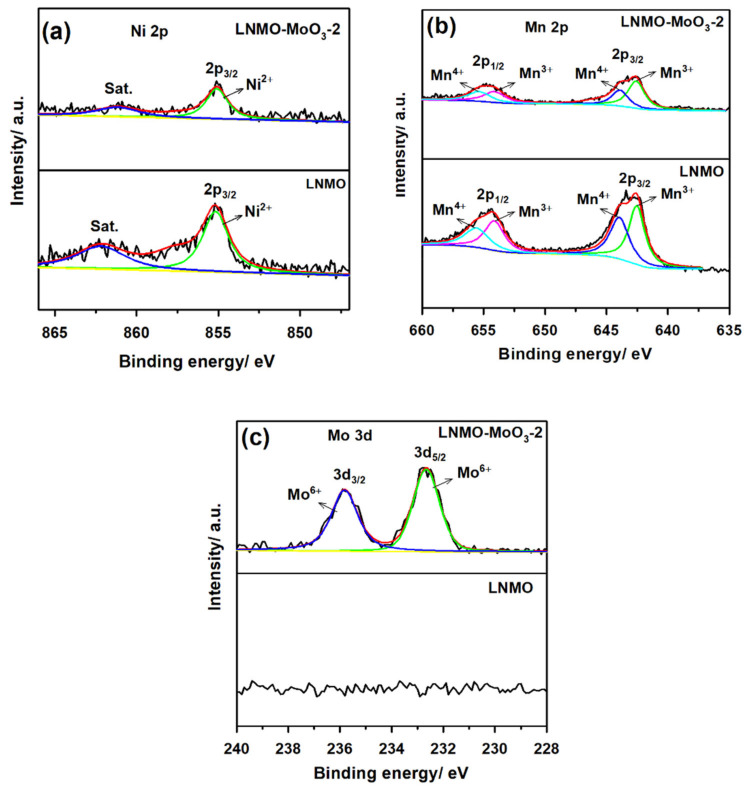
Ni 2p (**a**), Mn 2p (**b**) and Mo 3d (**c**) XPS spectra of the LNMO and LNMO-MoO_3_-2 samples.

XPS was conducted to investigate the surface structure of LNMO before and after MoO_3_ coating. The results obtained from the XPS spectra confirmed the presence of several elements, including Li, Ni, Mn, and O, in LNMO and LNMO-MoO_3_-x. The signals corresponding to the Mo elements were clearly observed for the MoO_3_ coating samples. The deconvoluted XPS peaks of Ni 2p and Mn 2p for LNMO-MoO_3_-2 were similar to those observed for LNMO, indicating that MoO_3_ coating did not affect the oxidation states of Ni and Mn (Figure 3a,b). The Ni 2p XPS peaks observed at 855.2 eV corresponded to Ni 2p_3/2_, which is in good agreement with those observed for Ni^2+^ [31]. The deconvoluted XPS peaks of Mn 2p split into two peaks, namely Mn 2p_3/2_ and Mn 2p_1/2_ peaks, with finding energies of the fitted peaks of 642.6 and 654.2 eV for Mn^3+^ and 644.0 and 655.5 eV for Mn^4+^, respectively [32]. Thus, a small quantity of Mn^3+^ was present in the LNMO and LNMO-MoO_3_-2 samples. The high-temperature calcinations of LNMO led to the facile induction of a slight oxygen deficiency. Thus, the presence of a small amount of Mn^3+^ in LNMO materials cannot be avoided [27,33]. Figure 3c displays the oxidation state of Mo in the LNMO-MoO_3_-2 sample. The peaks observed at binding energies of 232.7 eV (Mo 3d_5/2_) and 235.8 eV (Mo 3d_3/2_) indicated the presence of Mo^6+^ [34,35]. By contrast, no obvious signal was observed in the pristine LNMO samples. The aforementioned results confirmed that the MoO_3_ layers had been coated onto the LNMO surface.

### 3.2. Electrochemical Characterization

**Figure 4:**
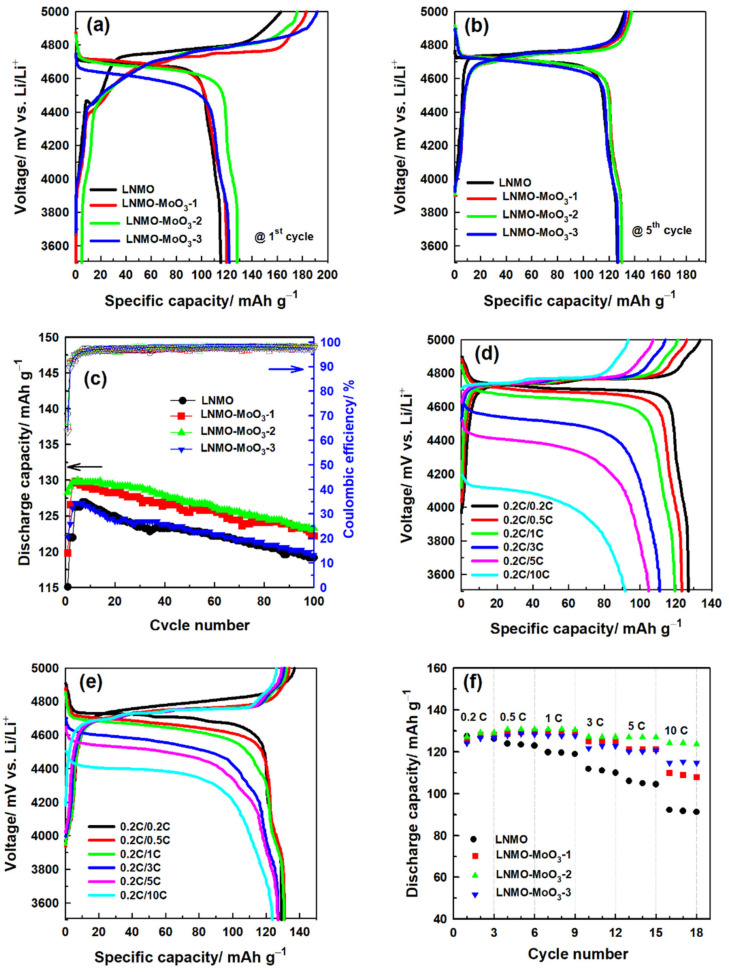
Charge–discharge curves of pristine LNMO and LNMO-MoO_3_-x electrodes at 0.1 C rate for (**a**) initial and (**b**) fifth cycles; (**c**) Cycling stability results of the pristine LNMO and LNMO-MoO_3_-x electrodes at 0.1 C for 100 cycles; Charge–discharge curves of pristine LNMO (**d**) and LNMO-MoO_3_-2 (**e**) electrodes at 0.2–10 C; (**f**) corresponding rate capability curves of the electrodes.

Figure 4a,b display the results of charge–discharge analysis of the initial and fifth cycles measured at a rate of 0.1 C (1 C = 147 mA g^−1^) for LNMO and LNMO-MoO_3_-x electrodes, with varied potential in the range of 3.5–5 V (vs. Li/Li^+^). The curves of all the samples consisted of a 4.7 V long plateau and a 4.0 V short heel plateau, which corresponded to the Ni^2+^/Ni^4+^ and Mn^3+^/Mn^4+^ redox couple, respectively. This phenomenon was also confirmed through cyclic voltammetry analysis. In this case, typical redox couples (Ni^2+^/Ni^4+^ and Mn^3+^/Mn^4+^) were observed in both electrodes (Figure S1). Charge–discharge analysis revealed the initial specific discharge capacities of 115, 120, 128, and 122 mAh g^−1^ for pristine LNMO, LNMO-MoO_3_-1, LNMO-MoO_3_-2, and LNMO-MoO_3_-3 samples, respectively, which indicated that a dense MoO_3_ coating layer can effectively increase the initial specific discharge capacities. The observed coulombic efficiencies at initial cycles were 70.7%, 67.2%, 65.4%, and 63.6% for LNMO, LNMO-MoO_3_-1, LNMO-MoO_3_-2, and LNMO-MoO_3_-3, respectively, By contrast, the initial coulombic efficiencies decreased, suggesting that the surface roughness of the materials affected the formation of the CEI layer. However, the electrodes exhibited the largest specific discharge capacities of 126, 128, 130, and 126 mAh g^−1^ in the fifth cycle, which corresponded to coulombic efficiencies of 95.4%, 95.9%, 94.7%, and 95.1%, respectively. Furthermore, after 100 cycles of testing at a charge–discharge rate of 0.1 C, the specific discharge capacities of pristine LNMO, LNMO-MoO_3_-1, LNMO-MoO_3_-2, and LNMO-MoO_3_-3 electrodes considerably decreased to 119, 124, 122, and 120 mAh g^−1^, respectively, followed by capacity retentions of approximately 94.4%, 94.9%, 94.6%, and 94.3%, respectively (Figure 4c). The aforementioned results indicate that the MoO_3_ coating layer could effectively suppress the HF (from the electrolyte) attack on the LNMO material and that the oxide coating layers could enhance long-term cycling stability [35].

Figure 4d,f and Figure S2a,b reveal the rate capabilities of the LNMO electrodes under ambient conditions at a fixed charge rate of 0.2 C, and various discharge rates in the range of 0.2–10 C for every third cycle. The results revealed that the LNMO-MoO_3_-2 electrode exhibited the best electrochemical performance, with specific discharge capacities of 129, 131, 131, 127, 127, and 124 mAh g^−1^ at rates of 0.2, 0.5, 1, 3, 5, and 10 C, respectively (Figure 4e), whereas the corresponding specific discharge capacities of the pristine LNMO electrode at the same discharge rate were only 127, 123, 120, 111, 105, and 92 mAh g^−1^, respectively (Figure 4d). Moreover, with an increase in rate, the LNMO-MoO_3_-1 and LNMO-MoO_3_-3 electrodes exhibited superior performance compared with that of the pristine LNMO electrode (Figure 4f). The rate profiles of pristine LNMO and LNMO-MoO_3_-2 electrodes were also tested at rates of 0.2 C–10 C and exhibited excellent reversibility (Figure S3). This proves that MoO_3_ coating of the LNMO surface enhanced the rate performance. This result is mainly attributed to the effective isolation of the cathode material from the electrolyte by the coated oxide layer. The spinel structure of LNMO maintains three-dimensional Li^+^ ion transition channels to reduce the polarization effect at high rates and accelerate the transport rate of Li ions, thereby enhancing the utilization of the cathode materials at high charge–discharge rates [36].

**Figure 5:**
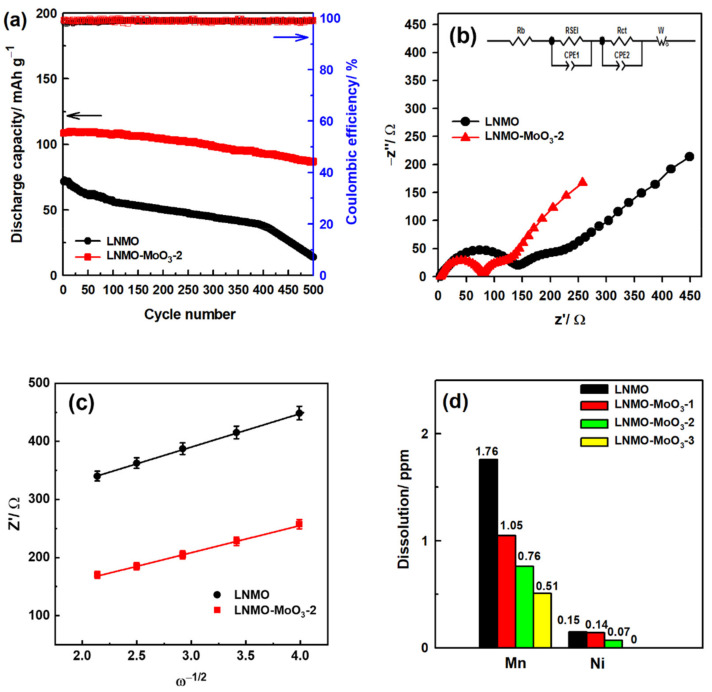
(**a**) The cycle-life of the pristine LNMO and LNMO-MoO_3_-2 electrodes for 500 cycles at 10 C rate; (**b**) EIS Nyquist plot and equivalent circuit model (inset) and (**c**) Z’ vs. ω^−1/2^ plot of the pristine LNMO and LNMO-MoO_3_-2 electrodes after 500 cycles at 10 C rate; the concentration of Mn and Ni ions dissolved in the organic electrolyte for LNMO and LNMO-MoO_3_-x electrodes stored at 60 °C for 7 days (**d**).

The aforementioned rate capabilities indicate that the LNMO-MoO_3_-2 electrode has superior electrochemical performance at rates of 0.5 to 10 C. We compared the LNMO and LNMO-MoO_3_-2 electrodes to observe their long-term cycle stability at higher rates. Figure 5a displays a stable discharge capacity for LNMO and LNMO-MoO_3_-2 electrodes after performing at a rate of 1/10 C for 500 cycles. The results indicated that the LNMO-MoO_3_-2 electrode exhibited an initial specific discharge capacity of 109 mAh g^−1^, which decreased to 87 mAh g^−1^ after 500 cycles with a capacity retention of approximately 80.1%. By contrast, the pristine LNMO electrode exhibited a lower specific discharge capacity (72 to 14 mAh g^−1^) and capacity retention (19.5%). However, the coulombic efficiencies of both the electrodes reached approximately 99%. The low discharge capacity and poor capacity retention of the LNMO cathode indicated that considerable Mn dissolution occurred in the pristine LNMO samples during the cycling process. As seen in Figure S4, the Mn (around 3.7–4.0 V) plateau at the 5th cycle is clearly visible with both LNMO and LNMO-MoO_3_ electrodes. Such a plateau is still observed in the LNMO-MoO_3_ electrode after the 500th cycles, but it is significantly reduced in the LNMO electrode after the 200th cycles. The results clearly confirm that the Mn dissolution was caused by the formation of HF by the decomposition of the electrolyte when the cell was operating at high voltage [37,38]. Regarding the alleviation of Mn dissolution through LNMO, this study demonstrated that MoO_3_ coating is an effective method for inhibiting the occurrence of side reactions and thereby improving the overall specific discharge capacity and cycle stability of high-voltage cathode materials.

To confirm the influence of MoO_3_ coating, we analyzed the cycled electrodes. The FE-SEM and HRTEM images of the pristine LNMO and LNMO-MoO_3_-2 electrode materials were obtained after 500 cycles at different rates between 1 and 10 C (Figure S5). The results revealed that the MoO_3_ coating layer effectively protected the LNMO cathode from HF attacks, whereas cracks in the surface were observed on the pristine electrode because of the side reaction between LNMO and electrolyte species. In addition, we examined the pristine LNMO and LNMO-MoO_3_ cathode after cycling testing with XRD analysis. As observed in Figure S6, the LNMO-MoO_3_ cathode had a much sharper lower angle change than the pristine LNMO. The MoO_3_ coating layer effectively protects the LNMO from electrolyte species (HF) and stabilizes the crystal structure of the LNMO during the high voltage operation. From the results, we confirmed that the MoO_3_ layer has a significant effect on controlling the dissolution of Mn and Ni in LNMO. Therefore, the MoO_3_ coating layer exhibited the best protection performance of the LNMO cathode materials at high-voltage operation of the battery system. Finally, we compared and summarized the rate capability and cycling performance of the MoO_3_-coated LNMO cathode material with other reported coating materials [20,22,23,39,40,41,42,43,44,45,46,47,48,49,50]. The comparison results can be seen in Table 1. Recently, Bai et al. [50] reported the effect of MoO_3_ coating on the LNMO spinel cathode by mixing the MoO_3_ powder with the LNMO cathode with the annealing process. Their report results show that the 2 wt.% MoO_3_ coated-LNMO is worse than our LNMO-MoO_3_-2 cathode. Our LNMO-MoO_3_-2 cathode showed excellent discharge capacity at high rates from 1 to 10 C. Furthermore, the resulting capacity retention at 10 C was considerably stable throughout the 500 cycles. Moreover, electrolyte decomposition is a concern during high-voltage operation. In this case, single oxides are a promising candidate for protective coating and can be extensively used in high-voltage LIBs.

**Scheme 1:**
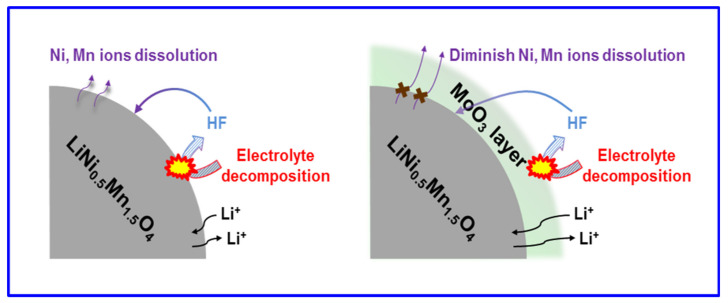
Schematic representation of MoO_3_-coated LNMO and the role of MoO_3_ as an effective coating layer on the cathode surface.

Electrochemical impedance spectroscopy (EIS) was performed after cycling LNMO-MoO_3_-2 and pristine LNMO electrodes at a rate of 10 C for 500 cycles (Figure 5b). Each EIS plot displays semicircles and straight lines, which indicate high- and low-frequency ranges, respectively. The related circuit model is provided in the inset of Figure 5b. Here, *R*_b_ refers to the bulk solution resistance, *R*_SEI_ corresponds to the impedance of Li^+^ ion diffusion over the surface of active materials, *R*_ct_ is the electron transfer resistance at the electrode/electrolyte interfaces, and CPE denotes the double-layer capacitance. The straight line (indicating low frequency) is associated with the Warburg impedance, which can be used to calculate Li^+^ ion diffusion (*D*_Li_^+^) in the bulk material. Diffusion can be calculated using the following Equation (3) [51]:

(3) $$D_{Li^{+}}=\frac{R^{2}T^{2}}{2A^{2}n^{4}F^{4}C_{Li}{}^{2}\sigma^{2}}$$

where *R* is the gas constant, *T* is the temperature, *A* is the electrode area, *n* is the electron transfer number, *F* is Faraday’s constant, *C*_Li_^+^ is the molar concentration of Li ions in the bulk material (*C*_Li_^+^ = 0.02378 mol cm^−3^) [52], and *σ* refers to the Warburg factor, which is calculated from the slope of the linear plot of *Z*’ versus *ω*^−1/2^ (Figure 5c). Parameters *R*_b_, *R*_SEI_, *R*_ct_, and *D*_Li_^+^ are summarized in Table 2. The *R*_b_ values of the pristine LNMO and LNMO-MoO_3_-2 electrodes were approximately 3.92 and 4.04 Ω, respectively. Moreover, the LNMO-MoO_3_-2 electrode exhibited lower *R*_SEI_, which indicates that the as-formed MoO_3_ coating layer considerably reduced the thickness of the formed R_SEI_ layer. Furthermore, the *R*_ct_ of the pristine LNMO and LNMO-MoO_3_-2 electrodes were 74.20 and 49.37 Ω, respectively. As compared to the initial stage cells that were cycled at 0.1 C for 5 cycles (Figure S7 and Table S1), the D_Li_^+^ of both LNMO and LNMO-MoO_3_-2 electrodes were decreased due to the increase in the R_SEI_ layer. However, the results revealed that the side reactions at the interface were markedly inhibited on the LNMO-MoO_3_-2 electrode by a MoO_3_ coating layer (Scheme 1). Therefore, the electrochemical performance of LNMO-MoO_3_-2 was superior to that of the pristine LNMO electrode.

The pristine *D*_Li_^+^ value of the LNMO (1.04 × 10^−14^ cm^2^ s^−1^) electrode was lower than that of the LNMO-MoO_3_-2 (1.59 × 10^−14^ cm^2^ s^−1^) electrode. The obtained result proved that MoO_3_ coating can facilitate fast Li^+^ ion intercalation and deintercalation kinetics in electrodes, and thereby enhance high-rate performance (Figure 4). To investigate the sensitivity of the spinel LNMO cathode material to the dissolution behavior of transition metal ions under high temperatures, we soaked the LNMO and LNMO-MoO_3_-x electrodes in an organic electrolyte solvent with an approximate ratio of 4 mg active materials to 5 mL of organic electrolyte at 60 °C for 7 days in a glove box environment. The dissolution of the metal ions from storage electrolytes was analyzed after processing. Figure 5d illustrates that the dissolved amounts of Mn and Ni ions gradually decreased with the increase in amount of MoO_3_ coating, indicating that the MoO_3_ coating layer can greatly suppress the dissolution of transition metals. By contrast, the pristine LNMO surface eroded because the generated HF directly attacked the exposed LNMO surface (Scheme 1).

**Figure 6:**
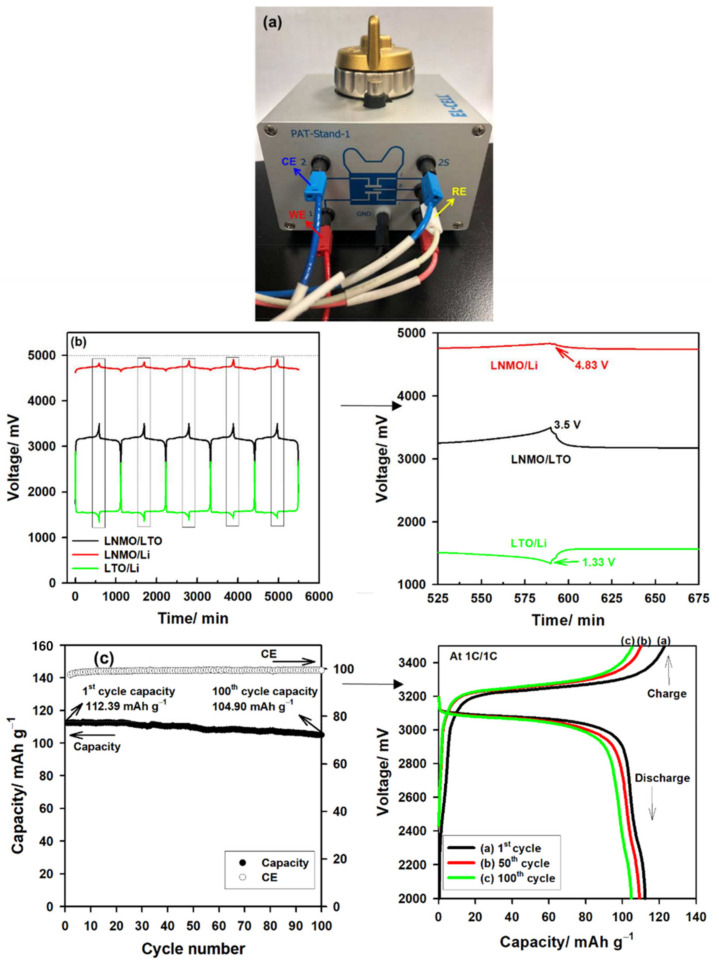
(**a**) Schematics of three-electrode battery system for full-cell test. (**b**) The charge–discharge curves of LTO-limited full cell at 0.1 C using three-electrode battery system with corresponding magnified curves (right). (**c**) The long-term cycling performance for LTO/LNMO full cell and its corresponding charge–discharge curves at 1 C/1 C for 100 cycles at 25 °C (right).

To demonstrate that the prepared LNMO cathode material can be used in a full cell, we assembled the LNMO/LTO full cell with a three-electrode configuration [53] (Figure 6a) to analyze its electrochemical behavior. In this three-electrode system, MoO_3_-coated LNMO was used as the working electrode, lab-made LTO [54] as the counter electrode and Li as the reference electrode. The battery testers comprising three individual channels were operated in sequence. Channel I was used to observe charge–discharge curves of the LNMO/LTO full cell, whereas channel II and channel III were used to observe the potentials of LNMO/Li and LTO/Li half cells, respectively. The cycling voltage range of the charge–discharge profiles of the cell varied from 2.0 to 3.5 V, as depicted in Figure 6b. A sharp increase in voltage was observed for the LNMO/LTO cell during charging, whereas the voltage of the LTO/Li cell decreased from 1.55 to 1.33 V, and that of the LNMO/Li cell increased to 4.83 V. This indicated that the Li^+^ ions were not fully detached from the LNMO samples, whereas the as-extracted Li^+^ ions had almost completely intercalated into the LTO sample surface. This confirms the transformation of Li_8a_[Li_1/3_Ti_5/3_]O_4_ spinel into [Li_2_]_16c_[Li_1/3_Ti_5/3_]_16d_O_4_ rock salt [55], as depicted in the magnified image on the right of Figure 6b. Figure 6c displays the long-term cycling of the LTO/LNMO full cell at 1 C/1 C for 100 cycles. The discharge capacity was initially 112 mAh g^−1^ and then decreased to 105 mAh g^−1^; the capacity retention and average columbic efficiencies were 93.34% and 99.3%, respectively.

Accordingly, the LTO-limited full-cell system can safely provide relatively low voltages for an LNMO cathode, even in its fully charged state. This is essential for reducing the electrolyte decomposition rate and improving the high-rate charge–discharge performance and superior cycling stability of LIBs. Other studies [24,25] of high-voltage operations have reported that the liquid electrolyte was highly exposed to electrochemical decomposition on delithiated transition metal oxide (such as LNMO, NCM, and Li-rich oxide) surfaces, resulting in the formation of unwanted passive layers. The passive resistive layer always hinders charge transfer across the oxide surface during the charge–discharge process. The proposed method of MoO_3_ coating on LNMO can considerably reduce the occurrence of side reactions, with a small increase in impedance (*R*_ct_ increases from 35 to 83 Ω after 100 cycles). Furthermore, MoO_3_ enhances the electrochemical performance of LTO/LNMO cells. This enhancement can be attributed to a cell design with a suitable negative/positive electrode capacity (N/P) ratio. In this study, the cell capacity was defined by the LTO anode, and the N/P ratio was below 1 (0.9), which reduced the charging capacity to more than 5 V. The use of cathode material is less here; this is due to the upper limit of charging potential below 5 V. However, capacity fading goes much faster when charging the N/P ratio above 1, where the cell capacity is controlled by the cathode instead of the LTO anode. It is also very difficult to control the power supply at 5 V without overcharging. This can trigger side products and electrolyte decay when high charges occur. To overcome this problem, we demonstrated the novel LiNi_0.5_Mn_1.5_O_4_ design by controlling N/P = 0.9. This can completely avoid charging capacity above 5 V. In this experiment, electrolyte decomposition decreased; therefore, the columbic efficiency of the LTO/LNMO full cell considerably improved to approximately to 99.3%. The performance of the proposed full cell was comparable to that reported by Ding [42], who demonstrated that an LTO capacity-limited full system could achieve superior electrochemical performance, and that an LTO-limited full-cell system can address the problem of overcharging. Therefore, our cell system has practical applications.

## 4. Conclusions

Spinel LNMO cathode materials coated with MoO_3_ layers were successfully prepared using solid-state reactions and the wet-chemical method. MoO_3_ coating did not affect the crystal structure of pristine LNMO materials. Furthermore, MoO_3_ could be uniformly deposited on the surface. The MoO_3_ coating layer played a key role in the electrochemical kinetics of LNMO; it reduced the electrode polarization, diminished the side reactions that occur at the interface of the 5 V cathode and electrolyte, and improved the charge-transfer reactions at the interface. Thus, MoO_3_-coated LNMO samples, particularly the LNMO-MoO_3_-2 sample, exhibited improved electrochemical performance. The MoO_3_ coating is a simple and effective method for enhancing the rate capability and cycling stability of spinel LNMO cathode materials, and can be used for large-scale commercial applications. We studied the electrochemical performance for the LTO/LNMO full cell. An LTO/LNMO full cell with a suitable negative/positive electrode capacity ratio (N/P ratio = 0.9) exhibited excellent performance and long-term cycling stability. The full-cell capacity was limited by the LTO anode, and the N/P ratio was 0.9, which markedly reduced the charge potential by more than 5 V. The MoO_3_-coated LNMO sample is an excellent coating candidate for high-voltage LIBs.

## Figures and Tables

**Table 1:** Compare the rate capability and cycling performance of the MoO_3_-coated LiNi_0.5_Mn_1.5_O_4_ cathode material with other reported coating materials.

Coating Materials	Rate Capability (mAh g^−1^ at C−Rate)	Capacity Retention (%@C−Rate)/Cycles	Ref.
CuO (3 wt.%)	126 at 2 C; 99 at 10 C	95.6 at 0.5 C/100	[20]
ZrO_2_ (1 wt.%)	~130 at 1 C; 128 at 10 C	85.6% at 40 C/1200	[22]
RuO_2_ (0.56 wt.%)	~118 at 1 C; 20 at 10 C	96.1% at 0.5 C/150	[23]
Li_2_SiO_3_ (0.1 wt.%)	~107 at 2 C	85.5% at 1 C/300	[39]
Polyaniline (1 wt.%)	120 at 1 C; 66 at 5 C	99.7% at 0.5 C/200	[40]
Al_2_O_3_ (0.5 wt.%)	101 at 5 C; 83 at 7 C	92.6% at 1 C/200	[41]
Li_4_Ti_5_O_12_ (LTO)	114 at 1 C; 91 at 10 C	93.6% at 0.5 C/100	[42]
V_2_O_5_	111 at 1 C; 52 at 5 C	96.6% at 1 C/100	[43]
Li_3_PO_4_–TiO_2_	~100 at 1 C; 60 at 5 C	81.2% at 0.5 C/300	[44]
Co_3_(PO_4_)_2_ (2.87 wt.%)	~125 at 1 C; 82 at 10 C	94.8% at 1 C/100 (55 °C)	[45]
MnPO_x_ (2 wt.%) NiPO_x_ (2 wt.%)	120 at 1 C; 100 at 10 C 128 at 1 C; 110 at 10 C	69% at 10 C/1000 82% at 10 C/1000	[46]
AlF_3_ (1 wt.%)	121 at 0.2 C	81.7% at 0.2 C/100 (55 °C)	[47]
YF_3_	95 at 1 C; 32 at 5 C	84% at 0.1 C/100	[48]
SiO_2_ (0.8 wt.%)	~120 at 1 C; 90 at 5 C	91.3% at 3 C/1000	[49]
MoO_3_ (2 wt.%)	~118 at 1 C; 96 at 2 C	96% at 0.2 C/100	[50]
MoO_3_ (2 wt.%)	131 at 1 C; 124 at 10 C	80.1% 10C/500	This work

**Table 2:** EIS fitting results for LNMO and LNMO-MoO_3_-2 samples.

Sample	*R*_b_/Ω	*R*_SEI_/Ω	*R*_ct_/Ω	*D*_Li_^+^/cm^2^ s^−1^
LNMO	3.92	139.79	74.20	1.04 × 10^−14^
LNMO-MoO_3_-2	4.04	76.40	49.37	1.59 × 10^−14^

## Data Availability

The data are available on reasonable request from the corresponding author.

## Supplementary Materials

The following supporting information can be downloaded at: https://www.mdpi.com/article/10.3390/nano12030409/s1, Figure S1: Cyclic voltammograms of LNMO and LNMO-MoO_3_-2 electrodes at a scan rate of 0.1 mV s^−1^. Figure S2: Charge–discharge curves of pristine LNMO-MoO_3_-1 (a) and LNMO-MoO_3_-3 (b) electrodes at 0.2–10 C. Figure S3: Rate profiles of pristine LNMO and LNMO-MoO_3_-2 electrodes at 0.2 C/0.2 C–10 C/10 C rate. Figure S4: Charge–discharge cycles of LNMO and LNMO-MoO_3_-2 electrodes. Figure S5: FESEM (a,b) and HRTEM (c,d) images of LNMO (left) and LNMO-MoO_3_-2 (right) electrodes after 1C/10 C rate performance for 500 cycles. Figure S6: XRD pattern of pristine LNMO and LNMO-MoO_3_-2 electrode in fresh and after 500 cycles at 10 C rate. Figure S7: (a) EIS and (b) Z’ vs. ω^−1/2^ plot of LNMO and LNMO-MoO_3_-2 electrodes after 5 cycles at 0.1 C rate. Table S1: EIS fitting results for LNMO and LNMO-MoO_3_-2 electrodes after 5 cycles at 0.1 C rate.
