# Supplementary material for: MoO3 Nanoparticle Coatings on High-Voltage 5 V LiNi0.5Mn1.5O4 Cathode Materials for Improving Lithium-Ion Battery Performance
Source: Nanomaterials (Basel). 2022 Jan 26;12(3):409. doi: 10.3390/nano12030409 (PMC8840174; doi:10.3390/nano12030409)
Supplement: Supplementary file 1 [file nanomaterials-12-00409-s001.zip › nanomaterials-1544125-supplementary.pdf]

nanomaterials

Article

MoO_3 Nanoparticle Coatings on High-Voltage 5 V $LiNi_{0.5}Mn_{1.5}O_4$ Cathode Materials for Improving Lithium-Ion Battery Performance

Zong-Han Wu [1], Jeng-Ywan Shih [2], Ying-Jeng James Li [1,2], Yi-De Tsai [1], Tai-Feng Hung [1], Chelladurai Karuppiah [1,*], Rajan Jose [3] and Chun-Chen Yang [1,2,4,*]

[1] Battery Research Center of Green Energy, Ming Chi University of Technology, New Taipei City 24301, Taiwan; smartindigoboy@gmail.com (Z.-H.W.); yjli@mail.mcut.edu.tw (Y.-J.J.L.); d1161p002@mail.mcut.edu.tw (Y.-D.T.); taifeng@mail.mcut.edu.tw (T.-F.H.)
[2] Department of Chemical Engineering, Ming Chi University of Technology, New Taipei City 24301, Taiwan; drexel@mail.mcut.edu.tw
[3] Nanostructured Renewable Energy Materials Laboratory, Faculty of Industrial Sciences and Technology, University Malaysia Pahang, Kuantan 26300, Malaysia; rjose@ump.edu.my
[4] Department of Chemical and Materials Engineering, and Green Technology Research Center, Chang Gung University, Taoyuan City 333, Taiwan
* Correspondence: kcdurai.rmd@gmail.com (C.K.); ccyang@mail.mcut.edu.tw (C.-C.Y.)

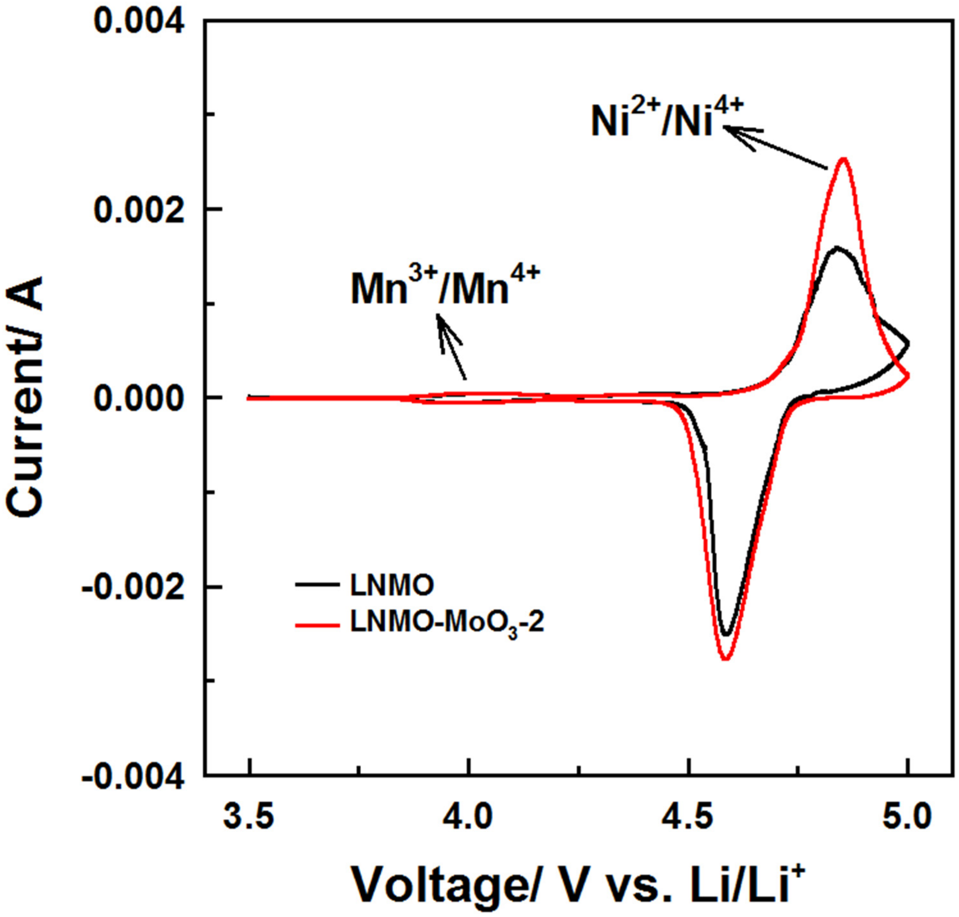

Figure S1. Cyclic voltamograms of LNMO and LNMO-MoO_3-2 electrodes at a scan rate of 0.1 mV s^{-1}.

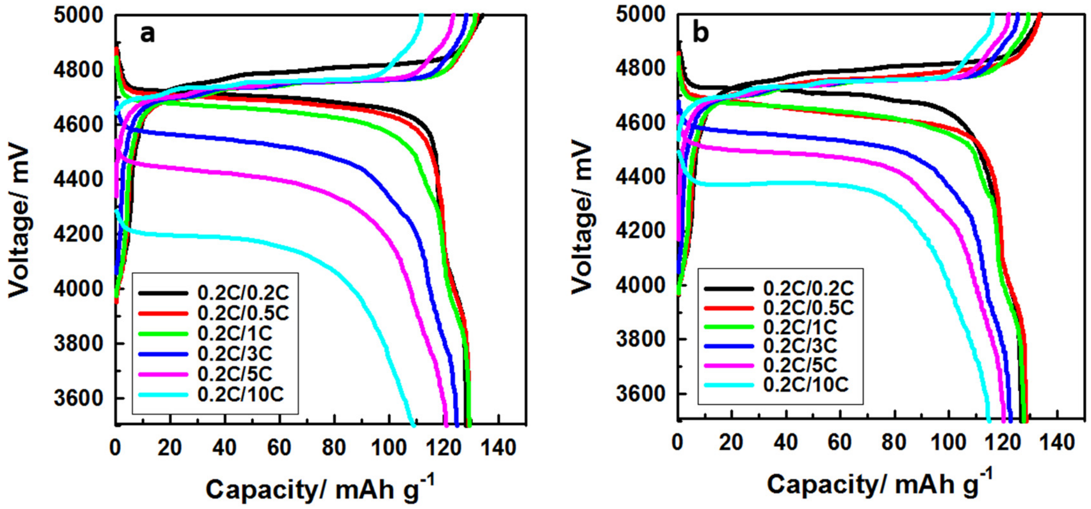

Figure S2. Charge-discharge curves of pristine LNMO-MoO_3-1 (a) and LNMO-MoO_3-3 (b) electrodes at 0.2–10 C.

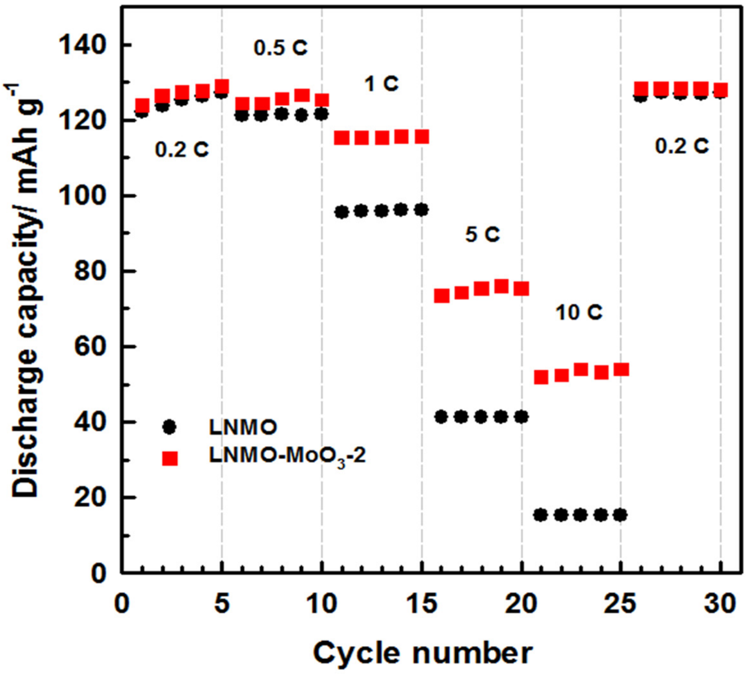

Figure S3. Rate profiles of pristine LNMO and LNMO-MoO_3-2 electrodes at 0.2C/0.2C–10C/10C rate.

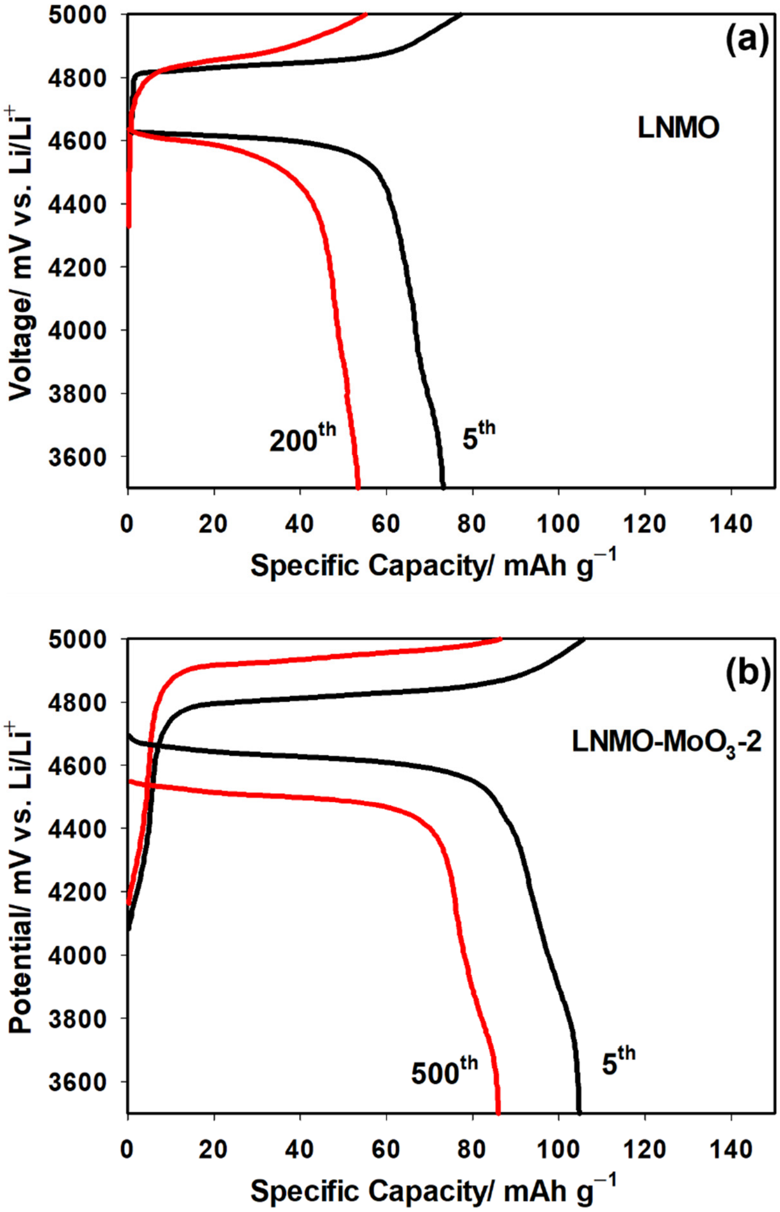

Figure S4. Charge-discharge cycles of LNMO and LNMO-MoO_3-2 electrodes.

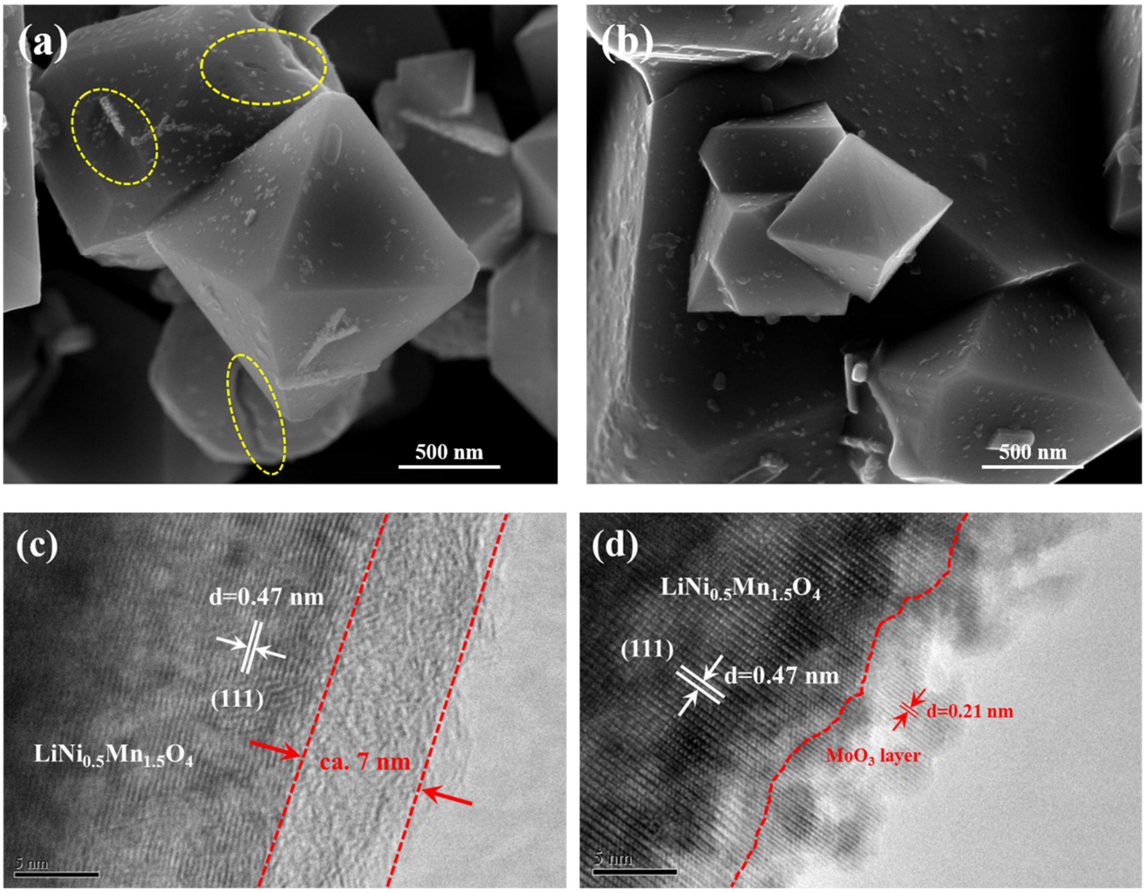

Figure S5. FESEM (a,b) and HRTEM (c,d) images of LNMO (left) and LNMO-MoO_3-2 (right) electrodes after 1C/10C rate performance for 500 cycles.

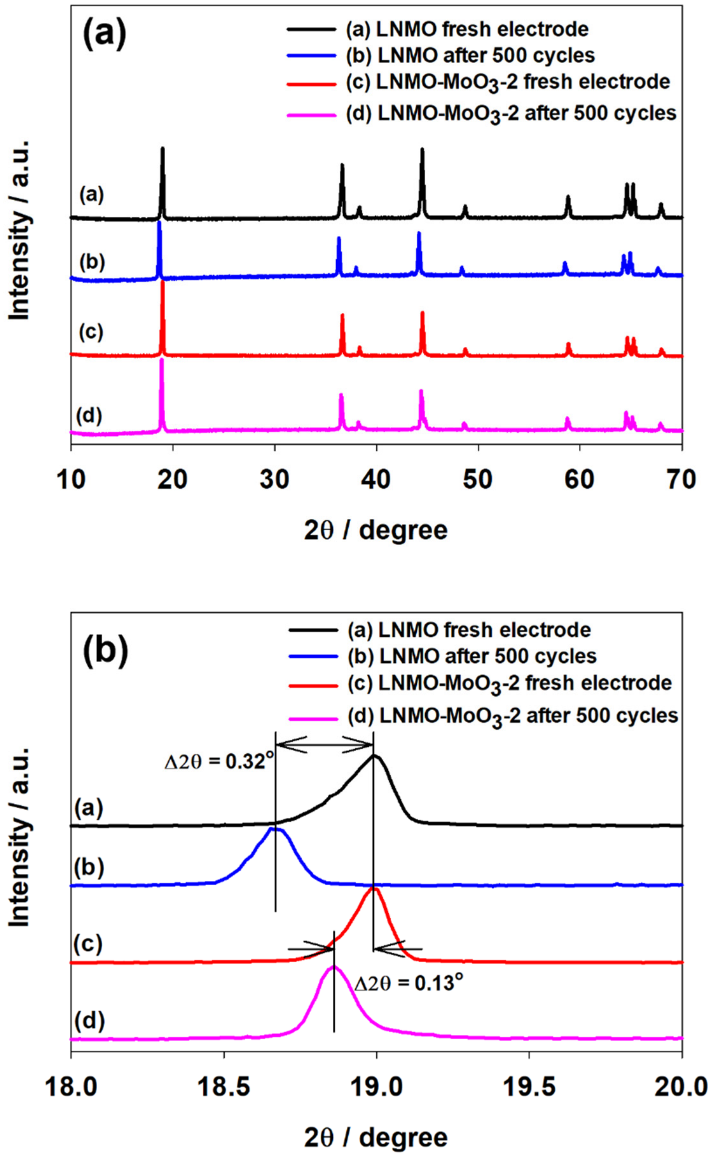

Figure S6. XRD pattern of pristine LNMO and LNMO-MoO_3-2 electrode in fresh and after 500 cycles at 10C rate.

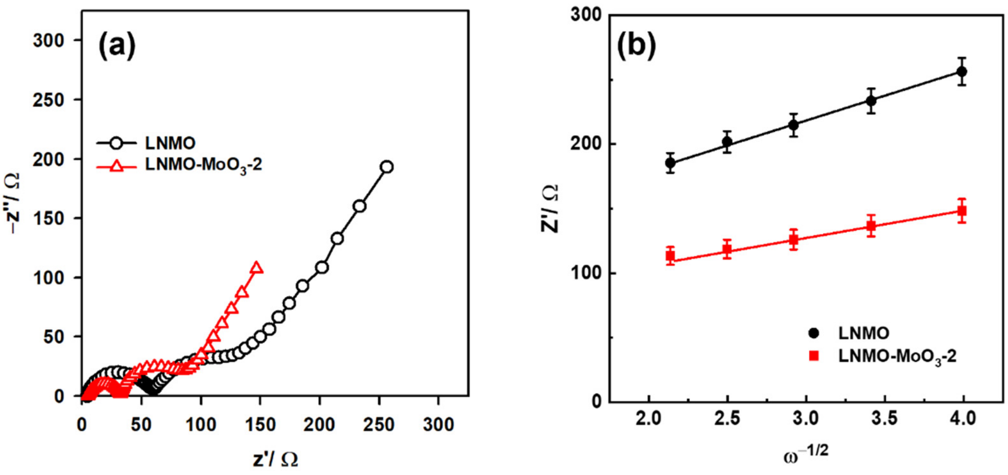

Figure S7. (a) EIS and (b) Z′ vs. $\omega^{-1/2}$ plot of LNMO and LNMO-MoO_3-2 electrodes after 5 cycles at 0.1C rate.

Table S1. EIS fitting results for LNMO and LNMO-MoO_3-2 electrodes after 5 cycles at 0.1C rate.

Sample	R_b/ Ω	R_{SEI}/ Ω	R_{ct}/ Ω	D_{Li^+}/ cm² s⁻¹
LNMO	4.30	55.13	67.08	2.52×10^{-14}
LNMO-MoO_3-2	5.13	27.23	52.74	9.61×10^{-14}
